# Review of Machine Learning in Predicting Dermatological Outcomes

**DOI:** 10.3389/fmed.2020.00266

**Published:** 2020-06-12

**Authors:** Amy X. Du, Sepideh Emam, Robert Gniadecki

**Affiliations:** ^1^Division of Dermatology, Faculty of Medicine and Dentistry, University of Alberta, Edmonton, AB, Canada; ^2^Information Services and Technology, University of Alberta, Edmonton, AB, Canada

**Keywords:** artificial intelligence, machine learning, dermatology, prediction, clinical outcomes

## Abstract

Artificial intelligence is a broad branch of computer science that has garnered significant interest in the field of medicine because of its problem solving, decision making and pattern recognition abilities. Machine learning, a subset of artificial intelligence, hones in on the ability of computers to receive data and learn for themselves, manipulating algorithms as they organize the information they are processing. Dermatology is at a particular advantage in the implementation of machine learning due to the availability of large clinical image databases that can be used for machine training and interpretation. While numerous studies have implemented machine learning in the diagnostic aspect of dermatology, less research has been conducted on the use of machine learning in predicting long-term outcomes in skin disease, with only a few studies published to date. Such an approach would assist physicians in selecting the best treatment methods, save patients' time, reduce treatment costs and improve the quality of treatment overall by reducing the amount of trial-and-error in the treatment process. In this review, we aim to provide a brief and relevant introduction to basic artificial intelligence processes, and to consolidate and examine the published literature on the use of machine learning in predicting clinical outcomes in dermatology.

## Introduction

Artificial intelligence (AI) is a broad branch of computer science that has garnered significant interest in the field of medicine because of its problem solving, decision making, and pattern recognition abilities. Machine learning (ML), a subset of AI, hones in on the ability of computers to receive data and learn for themselves, manipulating algorithms as they organize the information they are processing. Dermatology is at a particular advantage in the implementation of ML due to the availability of large clinical image databases that can be used for machine training and interpretation. In fact, studies have already demonstrated the successful use of ML in classification and diagnosis of skin diseases, such as skin cancer (1, 2), eczema (3), psoriasis (4), onychomycosis (5) at a performance level equal or superior to board-certified dermatologists.

While numerous studies have implemented ML in the diagnostic aspect of dermatology (6), less research has been conducted on the use of ML in predicting long-term outcomes in skin disease, with only a few studies published to date. In an era of personalized medicine, there is a push toward a data-driven approach allowing for accurate prediction of long-term clinical outcomes for individual patients (7–9). Such an approach would assist physicians in selecting the best treatment methods, save patients' time, reduce treatment costs and improve the quality of treatment overall by reducing the amount of trial-and-error in the treatment process (8).

Machine learning techniques are very good at managing large amounts of high-level data from patient databases, such as electronic medical records, and are often able to detect sophisticated data patterns that traditional statistical methods are unable to delineate (7–9). These approaches have been used with increasing success to predict patient prognoses in many other areas of medicine, such as the risk of readmission after hospital discharge (10), cancer progression (11), diabetic complications (12, 13), cardiovascular mortality (14), and many others (15–17).

In this review, we aim to provide a brief and relevant introduction to basic AI processes, and to consolidate and examine the published literature on the use of ML in predicting clinical outcomes in dermatology.

## Brief Overview of Principles in Artificial Intelligence

**Figure 1:**
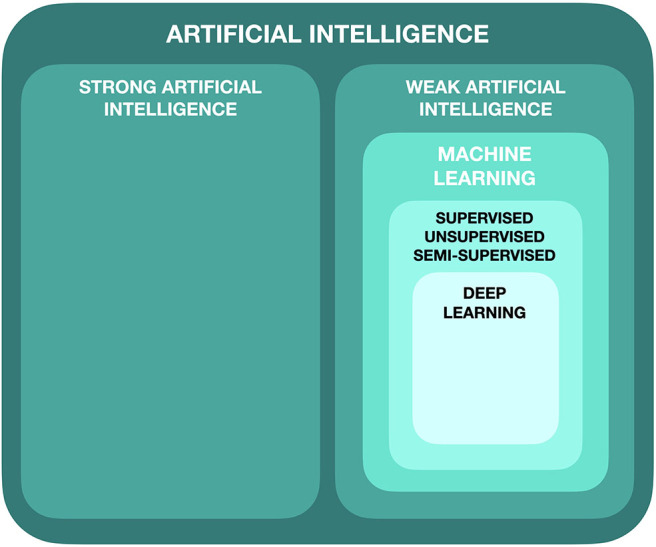
Subdivisions of artificial intelligence.

Artificial intelligence can be subdivided in a number of ways, but in its simplest form, it can be broken into two main categories: strong AI and weak AI (Figure 1).

Strong AI refers to a programmed machine that takes on human-level cognition, with the capacity for consciousness, self-awareness and ethical decision-making (18). The machine has the competence to learn, on its own, to simultaneously conduct a number of complex tasks, and the capability of learning more based on what it already knows (19). Currently, strong AI does not exist outside the realm of science fiction.

Weak AI, on the other hand, does currently exist and is the process by which we train a machine to complete a specific, designated task. The machine simply acts upon and is bound by the rules and algorithms that are set for it. It does not have the capacity, unlike strong AI, to think and act beyond those parameters (19).

### Machine Learning

Machine learning is a subdivision of AI in which algorithmic models are trained to perform specific tasks by recognizing and learning patterns from the data it sees, rather than through explicit computer programming by a human expert. This process can be categorized as supervised, semi-supervised or unsupervised, with the most common method being supervised (20).

Supervised learning occurs when the algorithm system gains experience through training with a labeled dataset, and is then expected to categorize a new, unfamiliar data point. For example, in the case of recognizing benign vs. malignant skin lesions, the computer system would be provided with many images of skin lesions that have already been labeled as either being benign or malignant. Once training with these images is completed, the algorithm would then be tested by being presented with novel, unlabeled images to classify as either being benign or malignant (21).

When no training dataset is available for the corresponding output data, it is known as unsupervised learning. Very much like supervised learning, the goal of this type of learning is to place input data into categories. The main difference is that, in unsupervised learning, the input data are not labeled, and therefore, the model aims to categorize data based on their inherent features. This method of machine learning allows us to take a more open-ended approach to learn about the underlying distribution of data that may have been missed otherwise (22).

Finally, a hybrid method of machine learning, known as semi-supervised machine learning, combines aspects of supervised and unsupervised learning. In this approach, a large amount of unlabeled inputs are combined with a small amount of labeled inputs in an effort to lessen the challenge of data labeling (22).

### Deep Learning

Deep learning is a further subdivision of ML and refers to a specific type of learning that involves the use of artificial neural networks (ANN) (23). It is often used for unsupervised learning, as it is capable of learning from data that is unstructured and unlabeled. It is able to detect patterns in datasets that it has not been previously trained on. Deep learning functions by imitating the neural connections made in the human brain and are connected in a network of nodes, forming multiple layers.

### Performance Analysis of Machine Learning Algorithms

In order to statistically evaluate the performance of learning approaches, and to best determine which approach predicts with the highest accuracy, machine learning algorithms are often assessed using the area under the curve receiver operating characteristic (AUC-ROC). This test quantifies how accurately a model is able to distinguish between categories, typically in medicine, “disease” vs. “no disease.” The ROC curve is plotted with the true positive rate on the y-axis against the false positive rate on the x-axis. The closer the AUC is to 1.0 for any given model, the better and more accurate the performance of that model (22).

## Methods

A literature search on Ovid MEDLINE® was conducted in January 2020 for papers published from 2000-2019, to focus on recently published literature. The database was searched with relevant keywords in combination with the Boolean operators “AND” and “OR.” The search included keywords from each of the following lists: dermatology, skin disease, skin cancer, psoriasis, or atopic dermatitis, AND artificial intelligence, machine learning, deep learning, or neural network, AND prediction, predicting, or outcome.

Inclusion criteria included: English language, original studies, and focusing on the prognostic utility of artificial intelligence/machine learning in dermatology (i.e., predicting outcomes, risk stratification, selection of best treatment). Exclusion criteria included: reviews, animal studies, case reports, systematic reviews, studies not published in English. Any studies focusing on the diagnostic utility of artificial intelligence/machine learning in dermatology, focusing on a different medical field, or not using machine learning methods were also excluded.

Our literature search yielded a total of 73 articles, among which 6 were deemed relevant to this review based on our inclusion and exclusion criteria.

## Applications of Machine Learning in Predicting Dermatological Outcomes

A total of six studies on the use of machine learning in predicting dermatological outcomes have been published to date (Table 1). One study focused on the risk of biologic discontinuation in psoriasis patients (24), two studies investigated the risk of developing non-melanoma skin cancer (25, 26), one study looked at response to wart treatment modalities (27), one study explored the complexity of reconstructive surgery after periocular basal cell carcinoma excision (28), and one final study examined the risk of developing chronic venous ulcers in patients with cardiovascular disease (29).

**Table 1:** Summary of literature on the use of machine learning in predicting dermatological outcomes.

**Study**	**Type of supervision**	**End point**	**Results**	**Software(s) utilized**	**Data used**
Emam et al. (24)	Supervised	Risk of discontinuation of biologic	AUC for predicted risk of discontinuation due to: Any reason 0.95 Lack of efficacy 0.91 Adverse event 0.88 Other reasons 0.80	Generalized linear model, support vector machine, decision tree, random forest, gradient boosted trees, deep learning	*n* = 681 psoriasis patients 13 clinically relevant features per patient
Wang et al. (25)	Semi-supervised	Risk of developing non-melanoma skin cancer	AUC 0.89 Sensitivity 83.1% Specificity 82.3%	Convolutional neural network (deep learning)	*n* = 9,494, 1,829 non-melanoma skin cancer patients, 7,665 random non-cancer controls 20 clinically relevant features per patient
Roffman et al. (26)	Supervised	Risk of developing non-melanoma skin cancer	AUC 0.81 Sensitivity 86.2% Specificity 62.7%	Artificial neural network (deep learning)	*n* = 462,630, 2,056 non-melanoma skin cancer patients, 460,574 non-cancer patients 13 clinically relevant features per patient
Khozeimeh et al. (27)	Supervised	Response to wart treatment method	Cryotherapy: AUC 0.902, accuracy 80% Immunotherapy: AUC 0.813, accuracy 98%	Fuzzy logic and adaptive network-based fuzzy inference system (ANFIS)	*n* = 180, 90 patients in cryotherapy group, 90 patients in immunotherapy group 7 clinically relevant features per patient in the cryotherapy group, 8 in the immunotherapy group
Tan et al. (28)	Supervised	Complexity of reconstructive surgery after periocular basal cell carcinoma excision	Naïve Bayesian Classifier: AUC 0.854 PPV 38.1% NPV 94.1% ADTree: AUC 0.835 PPV 31% NPV 97%	Decision table, Bayesian, tree-based methods, multivariate logistic regression, nearest neighbor classifier, support vector machine	*n* = 156 periocular BCC patients 7 clinically relevant features per patient
de Franciscis et al. (29)	Supervised	Risk of developing chronic venous ulcers in patients with chronic venous disease	CVU group level of risk 32.38 ± 7.19% Non-CVU group level of risk 8.34 ± 3.38%	Fuzzy logic to stratify CVD patients into CVU and non-CVU groups	*n* = 77, 40 patients with CVU, 37 patients without CVU 27 clinically relevant features

Five of the six studies used a supervised approach of machine learning in their training and validation. Wang et al. (25) used a semi-supervised approach. Generally, the results of each study were presented with varying outcomes, but AUC was reported as the primary outcome in five of the six studies. Other outcomes reported included sensitivity and specificity (25, 26), accuracy (27), and positive and negative predictive values (28). Franciscis et al. reported outcomes in the form of “level-of-risk” (29). We are unable to directly compare the outcomes of all studies as the methodology of the studies vary.

Emam et al. considered seven different modeling techniques in evaluating a dataset of 681 psoriasis patients to determine which learner performed best in terms of accuracy, interpretability and runtime to predict risk of biologic discontinuation. Thirteen clinically relevant features per patient were analyzed. The Generalized Linear Model (GLM) outperformed the six other models that were tested. The AUC for predicted risk of discontinuation due to any reason was found to be 0.95, lack of efficacy was 0.91, adverse event was 0.88, and other reasons was 0.80 using the GLM (24).

Wang et al. and Roffman et al. used convolutional neural networks (CNN) (25) and artificial neural networks (ANN) (26), respectively, to delineate the risk of developing non-melanoma skin cancer. Both approaches are branches of deep learning and make use of the algorithm's ability to extract important classifying information at each node of a network of data. Both studies included data from non-melanoma skin cancer patients as well as an abundance of data from non-cancer patients. The system by Wang et al. analyzed data from a total of 9,494 patients, using 20 clinically relevant features per patient, and reported higher outcomes (AUC 0.89, sensitivity 83.1%, specificity 82.3%) than Roffman et al., which analyzed data from a total of 462,630 patients, using 13 clinically relevant features per patient (AUC 0.81, sensitivity 86.2%, specificity 62.7%).

Two studies used fuzzy rule-based systems to stratify patients into groups. Fuzzy logic is a flexible mathematical system that can model non-linear functions with arbitrary meaning. It is a system that very closely models human thinking and is able to handle a great degree of uncertainty (30). Khozeimeh et al. (27) aimed to predict patient responses to two wart treatment modalities: cryotherapy and immunotherapy. Important clinically relevant features were extracted from the dataset using the Apriori algorithm and converted into fuzzy rules for each group. Data from a total of 180 patients, 90 in each group, were analyzed. Seven fuzzy rules were generated for the cryotherapy group and eight fuzzy rules were generated for the immunotherapy group. The resulting AUC of the cryotherapy and immunotherapy datasets was 0.902 and 0.813, respectively. The accuracy of both datasets was 80 and 98%, respectively.

Franciscis et al. (29) also used fuzzy logic to stratify the risk factors for developing chronic leg ulcers in patients in patients living with chronic venous disease (CVD). Data from seventy-seven CVD patients, 40 patients with active ulceration, 37 without, was analyzed. Twenty-seven clinically relevant features were generated for each patient. Results of the study were reported as risk scores, with the group of CVD patients with active venous ulceration being 32.38 ± 7.19%, and the group of CVD patients without active venous ulceration being 8.34 ± 3.38%.

Finally, Tan et al. (28) considered ten machine learning algorithms to determine the most predictive model for surgical complexity post-periocular basal cell carcinoma (BCC) excision. Data from 156 periocular BCC patients was analyzed, with seven clinically relevant features per patient. The most predictive model was Naive Bayesian classifier, with an average AUC of 0.854, and positive (PPV) and negative predictive values (NPV) of 38.1 and 94.1%, respectively. The second-best model was Alternating Decision Tree, achieving an AUC of 0.835, PPV of 31% and NPV of 97%.

## Discussion

Our review summarizes the current literature exploring the use of machine learning in predicting various dermatological outcomes. All studies conducted on this topic thus far have demonstrated promising outcomes. During a time where precision medicine is a focus of many clinicians, ML techniques provide a method for dermatologists to more accurately predict the clinical outcomes and prognoses of their patients in a variety of skin conditions.

When compared to traditional statistical methods, which focuses on inference, ML methodology focuses more on prediction (31). This is to say that ML methods aim to anticipate future behavior, rather than just drawing associations between data. ML is also particularly useful when looking at complex and detailed datasets with a large number of input variables. In fact, a larger sample size allows ML algorithms to better make associations within the data and thus, form more accurate outputs (32). Traditional statistical methods were designed to be most accurate and successful with a small to moderate number of input variables. As the number of inputs increases, the statistical models tend to become less precise.

While there are many benefits to the implementation of ML in a clinical dermatology setting, it is critical to discuss potential limitations to its implementation as well. One area of concern is the quantity of data required to operate ML algorithms. Some large dermatology patient registries do exist (33), however, there will need to be significantly more national and international collaboration to ensure that there is comprehensive coverage of dermatologic data.

Another limitation is the lack of ability for human operators to explain how ML algorithms make their conclusions. While there are methods to assess an algorithm's performance, there is no way to rationalize its decision. As such, they are often called “black box” technology (34). In this case, interpretation of the ML results by an experienced clinician is of utmost importance.

## Future Directions

Although early studies assessing the prognostic value of machine learning in dermatology have demonstrated promising outcomes, further research is needed. To address whether the use of ML in predicting outcomes is truly a worthwhile avenue for clinicians to explore, prospective randomized clinical trials are needed.

## Conclusion

Machine learning is a quickly advancing field in medicine and can be of great utility to clinicians in the near future, particularly in predicting the prognoses of complex dermatological conditions. As this technology advances, dermatologists will need to develop a foundational understanding of how it works and when it should be appropriately used in their clinical practice.

## Author Contributions

AD, SE, and RG have all provided substantial contributions to the conception and design of the work. AD drafted the work, SE and RG revised it critically for important intellectual content. All authors provide approval for publication of the content and agree to be accountable for all aspects of the work in ensuring that questions related to the accuracy or integrity of any part of the work are appropriately investigated and resolved.

## Conflict of Interest

The authors declare that the research was conducted in the absence of any commercial or financial relationships that could be construed as a potential conflict of interest.
